# Genome Sequence of a Freshwater Low-Nucleic-Acid-Content Bacterium, Betaproteobacterium Strain CB

**DOI:** 10.1128/genomeA.00135-13

**Published:** 2013-04-18

**Authors:** Zhenyu Hao, Lei Li, Jie Liu, Yan Ren, Lei Wang, Mark Bartlam, Thomas Egli, Yingying Wang

**Affiliations:** Key Laboratory of Pollution Processes and Environmental Criteria (Ministry of Education), Tianjin Key Laboratory of Environmental Remediation and Pollution Control, College of Environmental Science and Engineering, Nankai University, Tianjin, Chinaa;; TEDA School of Biological Sciences and Biotechnology and Tianjin Key Laboratory of Microbial Functional Genomics, Nankai University, Tianjin Economic-Technological Development Area; (TEDA), Tianjin, Chinab;; State Key Laboratory of Medicinal Chemical Biology & College of Life Sciences, Nankai University, Tianjin, Chinac;; Environmental Microbiology, Eawag, Swiss Federal Institute for Aquatic Science and Technology, Dübendorf, Switzerlandd;; Institute of Biogeochemistry and Pollutant Dynamics; (IBP), ETH Zürich, Zürich, Switzerlande

## Abstract

Betaproteobacterium strain CB is a typical minute freshwater bacterium, representing the small-cell bacteria that are numerically dominant in most freshwater environments. The genome of betaproteobacterium CB consists of a circular 2,045,720-bp chromosome, and the information we report will provide insights into the mechanisms underlying its survival and ecological function.

## GENOME ANNOUNCEMENT

Low-nucleic-acid-content (LNA) bacteria are widely distributed in aquatic environments and constitute numerically a large fraction of microbial communities (1, 2). Very little is known about these ubiquitous, small bacteria, particularly their ecological functions and metabolic mechanisms (3). Betaproteobacterium strain CB is a typical LNA bacterium possessing a distinctive small biovolume (<0.05 µm^3^), close to the size limit for free-living cells, and the ability to grow at low nutrient concentrations but not in conventional rich medium. Definitions in the available literature indicate the isolate should be a typical “obligate oligotroph” and an “ultramicrobacterium” (2, 3). The most comprehensive understanding of oligotrophs and ultramicrobacteria comes from the facultative oligotrophic bacterium *Sphingopyxis alaskensis* (4, 5). However, information on obligate oligotrophic bacteria is very limited. Here we present the whole-genome sequence of betaproteobacterium CB, with the aim of unraveling the mechanisms underlying its survival and ecological function. Strain CB was cultured by an unconventional cultivation approach, namely, using sterilized natural freshwater as a cultivation medium and monitoring growth by flow cytometry (2, 6). The whole betaproteobacterium CB genome was sequenced using Solexa paired-end sequencing technology. Genomic libraries were constructed containing 500-bp paired-end (PE) and 3-kb mate-pair (MP) inserts to generate 1,897,661,000 bp of hybrid data, assembled into 10 large contigs and providing 927.6-fold coverage of the genome. The reads, assembled into 46 contigs utilizing *de novo* assembly, were used to construct 10 scaffolds. Interscaffold and intrascaffold gaps were filled by sequencing PCR products using an ABI 3730 capillary sequencer. An error rate of less than 1 in a 10-kb sequence was achieved for the complete genome through sequence assembly and quality assessment using the Phred/Phrap/Consed software package (7, 8). The complete sequence was analyzed using Augustus, GlimmerHMM, SNAP, and GeneMark for protein-coding genes, Rbsfinder (http://www.tigr.org) for the ribosomal binding site, and tRNAscan-SE (9) for the tRNA. The functions of predicted protein-coding genes were annotated through comparisons with the NCBI nonredundant (NR) and Swiss-Prot protein databases from the European Bioinformatics Institute.

The genome consists of a circular 2,045,720-bp chromosome having no plasmid, with a G+C value of 46.09%. The chromosome contains 1 16S-23S-5S rRNA operon and 36 tRNA genes. The genome carries 2,112 putative protein-coding genes, encoding 188 transmembrane proteins and 1,924 non-transmembrane proteins. The majority of functional genes are involved in basic metabolism, protein translation, and DNA replication.

Strain CB belongs to the genus *Polynucleobacter* on the basis of 16S rRNA gene sequencing and genetic comparison with the strain *Polynucleobacter* sp. QLW-P1DMWA-1 (accession no. NC_009379.1). However, this type of strain is conventionally cultivable on rich media, suggesting that this genus is rather shallow and harbors many physiologically different organisms. In summary, we have sequenced the whole genome of a model LNA obligate oligotrophic ultramicrobacterium. To our knowledge, this first genome sequence for freshwater LNA bacteria will provide new insight into the physiological mechanisms of oligotrophic bacteria and their ecological roles.

### Nucleotide sequence accession number. 

The draft genome sequence of betaproteobacterium CB (10 large contigs) has been assigned GenBank accession no. CP004348.
